# Accessibility to biologics and its impact on disease activity and quality of life in patients with rheumatoid arthritis in Kuwait

**DOI:** 10.1007/s10067-020-05444-2

**Published:** 2020-10-12

**Authors:** Adeeba Al-Herz, Khuloud Saleh, Adel Al-Awadhi, Waleed Al-Kandari, Eman Hasan, Aqeel Ghanem, Mohammed Hussain, Yaser Ali, Ebrahim Nahar, Ahmad Alenizi, Sawsan Hayat, Fatemah Abutiban, Ali Aldei, Hebah Alhajeri, Naser Alhadhood, Husain Bahbahani, Hoda Tarakmeh, Khaled Mokaddem, Ahmad Khadrawy, Ammad Fazal, Agaz Zaman, Ghada Mazloum, Youssef Bartella, Sally Hamed, Ramia Alsouk, Ahmed Al-Saber, Kuwait Registry for Rheumatic Diseases (KRRD)

**Affiliations:** 1 Al-Amiri Hospital, Kuwait City, Kuwait; 2 Al-Farwaniya Hospital, Sabah Al-Nasser, Farwaniya Governorate Kuwait; 3 Department of Medicine, Faculty of Medicine, Kuwait University, Jamal Abdul Nasser St, Kuwait City, Kuwait; 4 Mubarak Al-Kabeer Hospital, Jabriya, Hawalli Governorate Kuwait; 5 Al-Jahra Hospital, Al Jahra, Jahra Governorate Kuwait; 6 Department of Mathematics and Statistics, University of Strathclyde, Glasgow, UK

**Keywords:** Biologics, DAS-28, Kuwait, Rheumatoid arthritis

## Abstract

**Objective:**

Biologics are indicated in rheumatoid arthritis (RA) in case of persistent high disease activity despite conventional disease-modifying anti-rheumatic drugs (cDMARDs) or patients with contraindications to cDMARDs or poor prognostic factors. The purpose of this study was to compare the prescription rates of biologics in Kuwaiti and non-Kuwaiti patients and to assess whether this had an impact on disease activity and quality of life in RA patients.

**Methods:**

Data were extracted from the Kuwait Registry for Rheumatic Diseases. Adult patients who satisfied the ACR classification criteria for RA from four major hospitals in Kuwait were evaluated from February 2013 through May 2018. The treatment agents, disease activity, and quality of life of Kuwaiti patients were compared with non-Kuwaiti patients.

**Results:**

A total of 1651 RA patients were included; 806 (48.8%) were Kuwaiti patients. Among Kuwaiti patients, 62.5% were on biologic drugs in comparison with 14% of non-Kuwaiti patients. In comparison with non-Kuwaiti patients, Kuwaiti patients had significantly lower numbers of swollen joints (*p* < 0.001) and disease activity score-28 scores (*p* = 0.02) and less steroid use (*p* < 0.001) yet a significantly higher health assessment questionnaire-disability index (*p* < 0.001). Regression analysis showed that DAS-28 scores were significantly associated with the treatment type (*p* < 0.001) and that nationality was significantly predictive of the treatment type (*p* < 0.001).

**Conclusion:**

In the setting of easy accessibility to treatment for Kuwaiti patients, biologics were prescribed by rheumatologists at a higher rate than for non-Kuwaitis. This may explain the lower disease activity and the lower rate of steroid use in Kuwaiti patients than non-Kuwaitis.

****Key points**:**

*• Significant discrepancies in the rates of prescribing biologic therapies between KP and NKP in Kuwait were observed*.

*• Several treatment outcomes were significantly better in the KP group than in the NKP group even after adjustment of confounding factors*.

*• The poor access to biologic therapies was suggested to limit the effectiveness of RA treatments in the NKP group*.

## Introduction

Rheumatoid arthritis (RA) is an autoimmune disease that affects 0.25 to 1% of the global population [1, 2]. In Kuwait, around 1% of the population is estimated to have RA [3]. Although the exact pathogenesis of RA remains unclear, several key players have been identified and targeted [4]. Several biological drugs have been approved by the FDA for the treatment of RA. These include drugs targeting tumor necrosis factor (TNF-α), interleukin (IL)–1, IL-6, B lymphocytes, and T lymphocyte co-stimulatory molecules. All these drugs have shown marked efficacy in clinical trials, especially in combination with methotrexate [5, 6].

In line with international guidelines [7, 8], the 2018 Kuwait Association of Rheumatology guidelines recommended the use of biologic therapy in (a) patients with early RA if they had persistent moderate to high disease activity despite conventional disease-modifying anti-rheumatic drugs (cDMARDs) and (b) patients with established RA, especially those with an inadequate response to cDMARDs or those with contraindications to cDMARDs, high disease activity, or poor prognostic factors. The guidelines further addressed the indications for TNF-inhibitor (TNFi)– or non-TNFi-based biologic therapy [9].

The population in Kuwait is 4,237,029 with 70% of the population being expatriates (non-Kuwaitis) [10]. The Ministry of Health provides all health services, including investigations and treatment to Kuwaiti patients at no cost. This also applies to all types of biologic treatments. Further, biologic therapy is provided to Kuwaiti patients (KP) within an average of a week after being evaluated by their treating rheumatologists. However, for non-Kuwaiti patients (NKP), the system is more complicated as the prescription has to go to a committee in a charity organization where the case is studied from medical and financial aspects. If approved, the charity organization will cover part of the cost depending on the patient’s budget. This usually takes 6 to 12 months until the medication is available, depending on how fast the paperwork is processed and the schedule of meetings to study the case. The 2018 Kuwait Association of Rheumatology guidelines were released in part to address the inequalities among KP and NKP in terms of access to biologics, lower disease activity, and better physical function [9].

This study is aimed at comparing the prescription rates of biologic drugs to KP and NKP and at assessing whether the process of providing biologic drugs to RA patients in Kuwait affects disease activity and quality of life in KP and NKP.

## Methods

### Study design

Data were extracted from the Kuwait Registry for Rheumatic Diseases (KRRD). The methods of data collection in this registry were described in a previous article [3]. Adult patients who satisfied the ACR classification criteria for RA [11] from four major hospitals (Amiri Hospital, Farwaniya Hospital, Mubarak Al-Kabeer Hospital, and Jahra Hospital) in Kuwait were evaluated from February 2013 through September 2019. Patients were divided into two groups: KP and NKP. Informed consent was obtained from all study subjects, and the study protocol was approved by the ethical committee at the Faculty of Medicine, University of Kuwait, and the Ministry of Health, Kuwait. All procedures performed involving human participants were in accordance with the ethical standards of the institutional committees and with the 1964 Helsinki declaration and its later amendments or comparable ethical standards.

### Data and outcome measurements

Data on the following parameters were collected: demographic data, treatment agents, and outcomes. These outcomes included the following: disease activity score-28 (DAS-28) [12], visual analog scale (VAS) pain, tender joints, swollen joints, erythrocyte sedimentation rate (ESR), C-reactive protein (CRP), physician and patient global assessment (GA) [13], and health assessment questionnaire-disability index (HAQ-DI) [14]. In the latter, patients reported the degree of experienced difficulty in performing common daily activities such as eating, dressing, and walking. The collected baseline data and outcomes were compared between the KP and NKP groups, using appropriate statistical methods.

### Statistical analysis

The normality of data was checked using Shapiro-Wilk’s test. Data were presented as count (%) or mean ± standard deviation. Pearson’s chi-squared and Fisher’s exact tests were used to compare between KP and NKP for the categorical variables. For numerical variables, a *t* test was used to compare the means between KP and NKP. To test the associations between the treatment type and DAS-28 scores between the two groups after controlling the confounding factors, the generalized linear model (GLM) was employed using a logistic regression approach. As patients’ characteristics differed across cohorts, a logistic regression model was used to adjust for baseline differences. The model adjusted for age, gender, rheumatoid factor (RF), smoking, anti-citrullinated peptide antibodies (ACPA), and disease duration. Adjusted odds ratios (ORs) and 95% confidence intervals (CIs) were used for logistic regression results. The statistical significance level was set at ≤ 0.05. All statistical analyses were conducted using STATA version Stata software, version 15.1 SE.

## Results

### Baseline characteristics

A total of 1651 RA patients with 10,282 hospital visits were enrolled in the current analysis, of whom 806 (48.8%) were Kuwaitis and 1024 (62%) were females. The mean age of all patients was 53.8 ± 12.5 years, the mean disease duration was 8.96 ± 6.73 years, 1174 (77.2%) had positive RF, and 864 (65.8%) had positive ACPA. Of all patients, 622 (37.7%) were on biologic therapy. In comparison with NKP, KP was significantly older, with longer disease duration, and had more females, with a higher frequency of comorbidities and secondary Sjogren’s syndrome, (*p* ≤ 0.001). However, NKP had a significantly larger number of smokers and ACPA positivity. Table 1 shows the baseline characteristics of the overall population and a comparison between KP and NKP in terms of these characteristics.

**Table 1:** Differences in demographic and basic characteristics between Kuwaiti and non-Kuwaiti patient groups

	Kuwaitis *N* = 806	Non-Kuwaitis *N* = 845	All Patients *N* = 1651	*p* value
Gender: female	578 (71.7%)	446 (52.8%)	1024 (62.0%)	< 0.001
Age in years	56.4 ± 13.2	51.4 ± 11.2	53.8 ± 12.5	< 0.001
RA duration	10.3 ± 7.53	7.61 ± 5.50	8.96 ± 6.73	< 0.001
Smoking	42 (7.23%)	85 (13.4%)	127 (10.4%)	0.001
Family history of a rheumatic disease	139 (25.0%)	80 (12.8%)	219 (18.6%)	< 0.001
Comorbidities	537 (66.6%)	347 (41.1%)	884 (53.5%)	< 0.001
2ry Sjogren’s	167 (24.6%)	81 (12.3%)	248 (18.6%)	< 0.001
Positive RF	538 (73.3%)	636 (80.9%)	1174 (77.2%)	0.001
Positive ACPA	371 (60.5%)	493 (70.4%)	864 (65.8%)	< 0.001
Positive ANA	180 (28.8%)	195 (28.3%)	375 (28.5%)	0.862

*ANA* antinuclear antibodies, *ACPA* anti-citrullinated peptide antibodies, *RA* rheumatoid arthritis, *RF* rheumatoid factor

### Treatment patterns

**Fig. 1:**
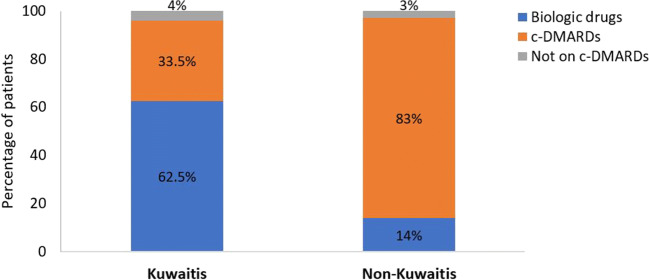
Treatment patterns among Kuwaiti and non-Kuwaiti patients

Among KP (*N* = 806), 503 (62.4%), 270 (33.5%), and 142 (18.6%) were on biologic therapies, cDMARDs, and steroids, respectively. Among NKP (*N* = 845), 119 (14.1%), 701 (83%), and 209 (26.4%) were on biologic therapies, cDMARDs, and steroids, respectively. A comparison between the two groups showed a significantly higher frequency of prescribing biological therapies in the KP group and a higher frequency of prescribing cDMARDs and steroids in the NKP group (*p* < 0.001). Figure 1 summarizes the frequencies of prescribing different therapeutic categories in the two groups.

### Treatment outcomes

**Fig. 2:**
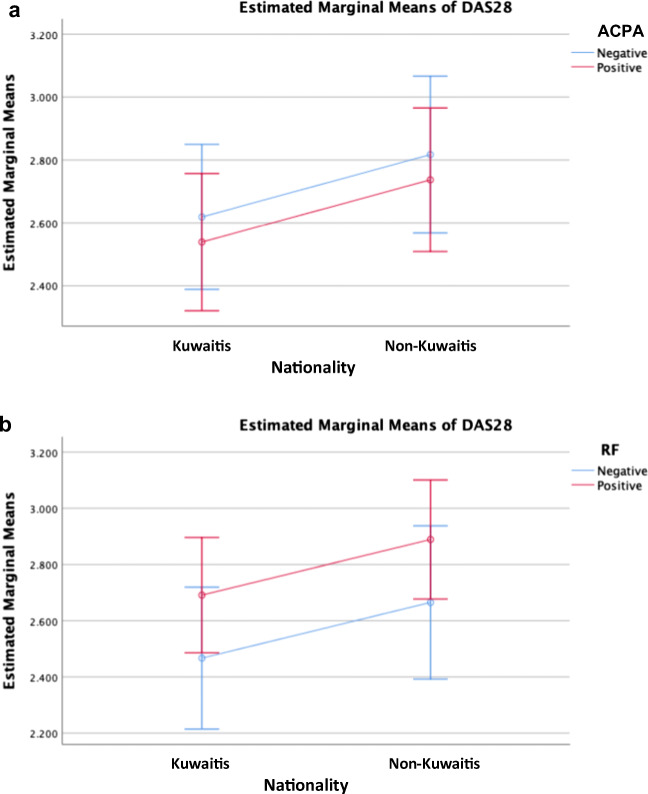
**a** Non-Kuwaiti patients who are rheumatoid factor positive have higher disease activity score-28 than their peers in the Kuwaiti group. **b** Non-Kuwaiti patients who are anti-citrullinated peptide antibody positive have higher disease activity score-28 than their peers in the Kuwaiti group

Among all the patients in the study, mean DAS-28 and HAQ-DI scores were 2.72 ± 1.28 and 0.99 ± 0.70, respectively. In comparison with NKP, KP had a significantly lower number of swollen joints (*p* < 0.001), lower DAS-28 scores (*p* = 0.02), and less steroid use (*p* < 0.001) yet a significantly higher HAQ-DI scores (*p* < 0.001). However, both groups were comparable in terms of the number of tender joints, VAS pain, ESR, CRP, physician GA, and patient GA (Table 2). NKP who are RF positive have higher DAS-28 than their peers in the KP group (Fig. 2a). Similarly, NKP who are ACPA positive have higher DAS-28 than their peers in the KP group (Fig. 2b).

**Table 2:** Differences in disease activity and quality of life between the KP and NKP groups

	Kuwaitis *N* = 806	Non-Kuwaitis *N* = 845	All Patients *N* = 1651	*p* value
Number of tender joints	2.98 ± 6.04	2.85 ± 5.29	2.91 ± 5.67	0.645
Number of swollen joints	0.42 ± 1.79	1.11 ± 2.97	0.78 ± 2.49	< 0.001
VAS pain	1.71 ± 2.53	1.59 ± 2.38	1.65 ± 2.46	0.311
ESR	28.0 ± 22.4	29.6 ± 22.9	28.8 ± 22.7	0.167
CRP	5.31 ± 4.77	5.75 ± 5.00	5.54 ± 4.89	0.115
HAQ-DI	1.07 ± 0.72	0.81 ± 0.63	0.99 ± 0.70	< 0.001
Patient GA	1.69 ± 2.49	1.72 ± 2.42	1.71 ± 2.45	0.798
Physician GA	1.05 ± 1.79	1.17 ± 1.84	1.11 ± 1.82	0.190
DAS-28	2.64 ± 1.29	2.79 ± 1.27	2.72 ± 1.28	0.020
Steroid use	79 (9.8%)	147 (17.4%)	226 (13.7%)	< 0.001

*p* values are significant at *p* < 0.05

*DAS-28* disease activity score-28, *VAS* visual analog scale, *ESR* erythrocyte sedimentation rate, *CRP* C-reactive protein, *GA* global assessment, *HAQ-DI* health assessment questionnaire-disability index

Analysis using the GLM showed that DAS-28 scores were significantly associated with the treatment type (beta = 0.18, *p* < 0.001, 95% C.I. [0.11, 0.25]) after controlling for the confounding factors including age, disease duration, gender, RF, smoking, and ACPA. In the same model, the interaction between DAS-28 and nationality was significantly predictive of the treatment type (beta = − 0.26, *p* < 0.001, 95% C.I. [− 0.38, − 0.13]). This means that the difference in DAS-28 scores between the two groups can be explained by the difference in the treatment type after controlling for the other confounders (Table 3).

**Table 3:** Generalized linear model using logistic regression

Constant	− 0.14 (− 0.58, 0.31)
DAS-28	0.18* (0.11, 0.25)
Age	− 0.02* (− 0.03, − 0.01)
Disease duration	− 0.07* (− 0.09, − 0.05)
Gender (male)	0.63* (0.45, 0.81)
RF (positive)	− 0.49* (− 0.67, − 0.30)
Smoking (yes)	− 0.52* (− 0.82, − 0.22)
ACCP (positive)	0.94* (0.78, 1.11)
Nationality (non-Kuwaitis)	3.32* (2.92, 3.72)
DAS-28 X nationality	− 0.26* (− 0.38, − 0.13)

*p < 0.001

## Discussion

Despite the availability of biological therapy options in the Middle East, there still is undertreatment of RA patients eligible for biologics. The suboptimal administration of biologics for RA patients in the Middle East and North Africa (MENA) is widely accounted for high medication costs and difficult access to therapies. Only 2% of RA patients in the MENA region received TNFi compared with 40% of RA patients in the USA [15]. A study in Doha revealed that 65% of Qatari RA patients receive biologics; however, only 15% of eligible non-Qatari patients receive biologics. Even with the high total health expenditure per capita, the main reason for this discrepancy was the lack of medical insurance for non-Qatari RA patients [16].

The current study showed significant discrepancies in the rates of prescribing biologic therapies between KP and NKP in Kuwait. Moreover, several treatment outcomes were significantly better in the KP group than in the NKP group even after adjustment of confounding factors including the number of swollen joints, mean DAS-28 scores, and the number of patients receiving steroids. Although these discrepancies did not affect all the efficacy variables in our study, however, the higher DAS-28 score reflected a higher disease activity in NKP in Kuwait. A retrospective analysis of 2282 patients showed that delays in the administration of biologic DMARDs could result from delayed approvals by healthcare insurers and the high cost of biologics paid through out-of-pocket expenses. These factors resulted in inadequate responses to therapy, which surprisingly did not affect the acceleration of treatment in that patient population [17]. The dose and duration of administered steroids were not of primary focus in our study; however, the well-known safety concerns of high doses or long durations of steroid administration, especially in RA patients, could have a clear impact on bone density and structure on the long term [18]. The combined impact of inadequate control of disease activity and safety concerns of high steroid rates indicates the need for rapid and realistic solutions to help change this situation in NKP. Despite the NKP having a higher disease activity, their HAQ-DI scores were lower than the KP. This may be explained by their tolerance to pain and their desire to overcome their functional limitations that may be caused by their disease. The majority of the NKP in Kuwait are of low socioeconomic status, and their need to work despite their disease is crucial for their living.

The sociodemographic impact on the management of RA was clearly observed in our study, represented in the NKP with mainly out-of-pocket expenditures for biological therapy. In 2017, a retrospective study in the USA analyzed medical expenditure data from 2009 to 2012. It highlighted that the average out-of-pocket expenditure was the highest for biological therapy being almost 28-fold more than the expenditure for DMARDs. The high cost of biologics negatively affected the patient adherence to medications especially in patients with no medical insurance or of low income [19]. In this study, the poor access to biologic therapies was documented to limit the effectiveness of RA treatments. These results are concordant with previous studies in the literature; however, the barriers against the accessibility to these drugs varied in different countries. For example, Orlewska et al. performed a study in Central and Eastern Europe and concluded that macroeconomic conditions and restrictive national guidelines were the main barriers against accessing biologic therapies [20]. In another study that surveyed 46 European countries, Putrik et al. found that patients in low–socioeconomic class countries had less access to biologic drugs, as well as worse RA treatment outcomes [21]. In Greece, Souliotis et al. reported that travel difficulties, late physician appointments, and drug shortage in hospitals increasingly limit the patients’ access to treatments [22]. Other factors such as the area of residence and low education class negatively impacted access to biologic agents. Moreover, elderly patients had less access to these drugs in other studies [23, 24].

To our knowledge, this is the first study to assess this association in the Middle East. Kuwait has an atypical demographic profile in which the majority of residents are non-Kuwaiti expatriates. The current laws allow free and immediate access to biologic drugs for KP, while it provides limited access to NKP. This allowed testing the impact of treatment accessibility within two diverse cohorts in the same country where the main difference is access to treatment. This hypothesis was further confirmed with logistic regression analysis after adjustment for other potential confounders.

The results of the current study support the recent guidelines of the Kuwait Association of Rheumatology that recommended addressing the inequality in accessing biologic therapies among KP and NKP in Kuwait. These results and recommendations should be conveyed to the policymakers and stakeholders to take effective actions against this problem. Another recommendation is not only to increase NKP access to biologic therapies but also to accelerate access to these drugs. Several studies have shown that delays in starting biologic therapy were associated with more advanced disease and worse RA treatment outcomes [17, 25].

Despite having a relatively large sample size, this study has some limitations. First, this is a retrospective study, inherently susceptible to different forms of bias, such as the recall bias with family history. Second, recording data from four different hospitals may introduce some heterogeneity related to inter-operator variability in outcome assessment and possibly different patient demographics. However, this adds to the strength of the wider generalizability of the generated results. Further, patients’ adherence to medications, the time from prescription to the application of biologics, and the side effects of drugs could not be extracted from the registry. These points should be assessed in future studies, not only in Kuwait but also in other Middle Eastern countries.

In conclusion, in the setting of easy accessibility to treatment, biologics were prescribed by rheumatologists at a higher rate than when approval was preceded by a strict and long protocol. This may explain the lower disease activity and the lower use of steroids in patients with rapid access to biologic treatment.

## Data Availability

All data generated or analyzed during this study are included in this published article.
